# *Atmiyata*, a community champion led psychosocial intervention for common mental disorders: A stepped wedge cluster randomized controlled trial in rural Gujarat, India

**DOI:** 10.1371/journal.pone.0285385

**Published:** 2023-06-08

**Authors:** Soumitra Pathare, Kaustubh Joag, Jasmine Kalha, Deepa Pandit, Sadhvi Krishnamoorthy, Ajay Chauhan, Laura Shields-Zeeman

**Affiliations:** 1 Centre for Mental Health Law and Policy, Indian Law Society, Pune, India; 2 Hospital for Mental Health, Ahmedabad, India; 3 Trimbos Institute (Netherlands Institute for Mental Health and Addiction), Utrecht, the Netherlands; 4 Faculty of Interdisciplinary Social Sciences, Utrecht University, Utrecht, the Netherlands; PLoS ONE, UNITED STATES

## Abstract

**Background:**

While effective lay-health worker models for mental health care have been demonstrated through efficacy trials, there is limited evidence of the effectiveness of these models implemented in rural LMIC settings.

**Aim:**

To evaluate the impact of a volunteer community-led intervention on reduction in depression and anxiety symptoms and improvement in functioning, and social participation among people living in rural Gujarat, India.

**Methods:**

Stepped-wedge cluster randomized controlled trial was used to assess the effectiveness of delivery of psychosocial intervention across 645 villages in Mehsana district of Gujarat, India between April 2017 and August 2019. The primary outcome was an improvement in depression and/or anxiety symptoms assessed using GHQ-12 at 3-month follow-up. Secondary outcomes were improvement in (a) depression and anxiety (Patient Health Questionnaire, (PHQ-9), Generalized Anxiety Disorder (GAD-7) & Self-Reporting Questionnaire-20 (SRQ-20); b) quality of life (EQ- 5D); c) functioning (WHO-DAS-12), and social participation (Social Participation Scale SPS). Generalized linear mixed-effects models were used to assess the independent effect of the intervention.

**Results:**

Out of a total of 1191 trial participants (608- intervention & 583-control), 1014 (85%) completed 3-month follow-up. In an adjusted analysis, participants in the intervention condition showed significant recovery from symptoms of depression or anxiety (OR 2.2; 95% CI 1.2 to 4.6; p<0.05) at the end of 3-months, with effects sustained at 8-month follow-up (OR 3.0; 95% CI 1.6 to 5.9). Intervention participants had improved scores on the PHQ-9 (Adjusted mean difference (AMD) –1.8; 95%CI -3.0 to -0.6), and SRQ-20 (AMD -1.7; 95%CI -2.7 to -0.6), at 3-months and PHQ-9, GAD-7, SRQ-20, EQ-5D and WHO-DAS at 8 months follow-up.

**Conclusion:**

Findings suggest that *Atmiyata* had a significant effect on recovery from symptoms of depression and anxiety with sustained effects at 8-month follow-up.

**Trial registration:**

**Trial registration details.** The trial was registered prospectively with the “Clinical Trial Registry in India” (registry number: CTRI/2017/03/008139).

## 1. Introduction

Although depression and anxiety disorders affect approximately 6% and 4% of the Indian population [1], there are limited services available to meet population needs, resulting in a treatment gap of 85% for common mental disorders (CMD) [1]. Reasons for the high treatment gap include a shortage of mental health professionals, public stigma towards mental illness, and limited accessible and affordable services [1]. Non-specialist health workers (NSHW) may be an option to improve access; however, a Cochrane review concluded that while interventions delivered by NSHWs and teachers showed promise in improving outcomes for depression, the evidence was of low or very low quality [2]. Recent studies in low-and middle-income countries (LMICs) have demonstrated the effectiveness of using non-specialist health workers to deliver psychosocial interventions for common mental disorders [3–5], particularly in primary health care settings. Scaling up and sustaining the implementation of these service delivery models remains challenging, as the context for implementation may not be favorable or able to take up additional mental health tasks [6]. Task sharing approaches may therefore need to build on existing community capacity to deliver some aspects of mental health care that complement efforts within the public service delivery system.

*Atmiyata* is a community-led intervention using non-specialized community volunteers for identification, support, treatment, and referral for persons with common and severe mental disorders [7]. The *Atmiyata* intervention aims to (i) reduce the treatment gap for common and severe mental disorders; (ii) improve mental health outcomes for people with common mental disorders; (iii) improve quality of life among people with mental disorders and (iv) improve access to social benefits for people with mental disorders. The intervention was previously piloted in 41 villages in Nashik district of the Indian state of Maharashtra from 2013–2015 [8]. Building upon the experience of the pilot study, the intervention was delivered at scale to a larger population in the state of Gujarat between 2017–2019.

This study presents findings on the effectiveness of the *Atmiyata* intervention on symptomatic improvement for people with depression and anxiety symptoms through a stepped-wedge cluster randomized controlled trial [9] in Gujarat, India.

## 2. Materials and methods

### 2.1 Study design

**Fig 1:**
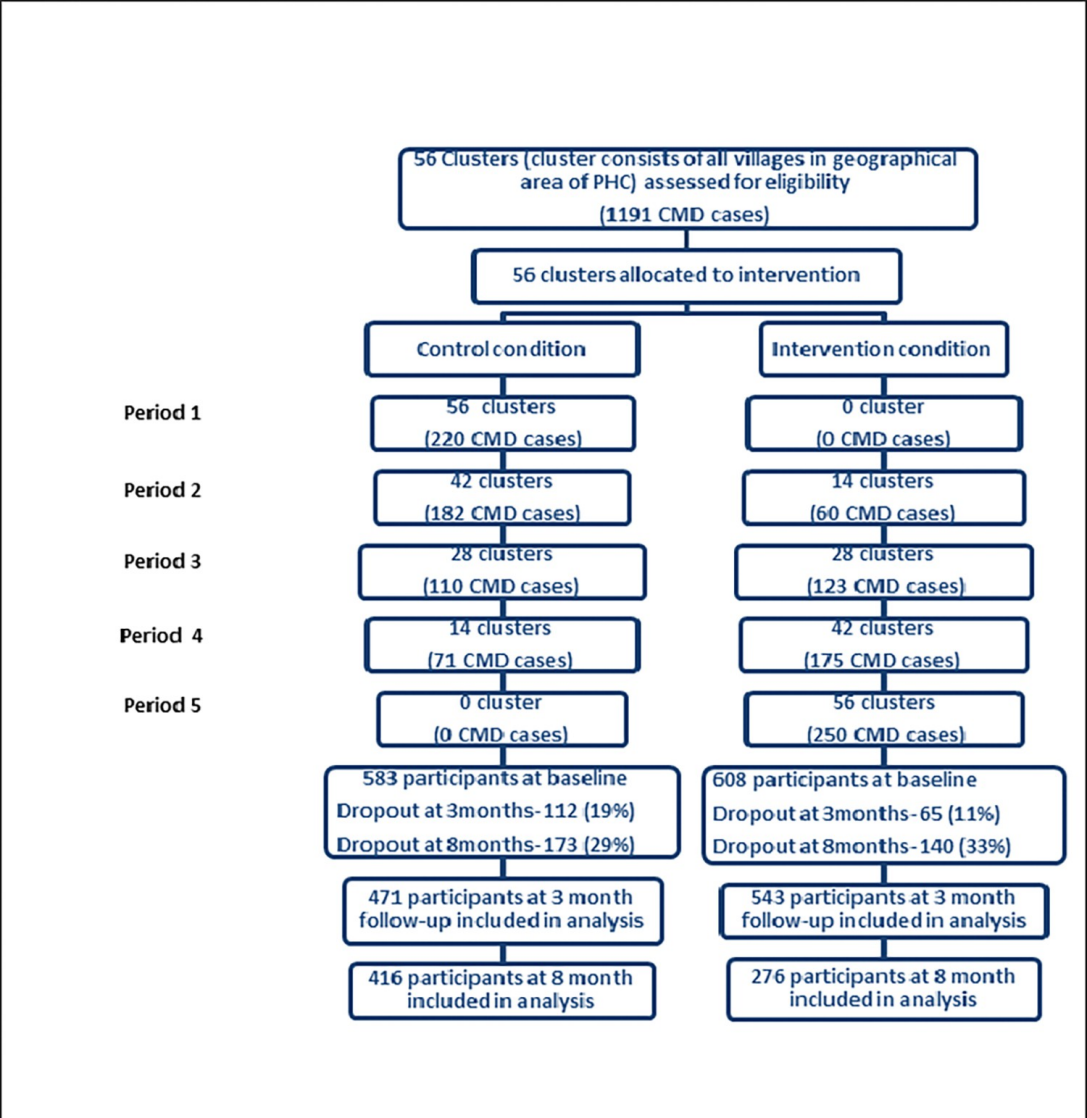
CONSORT flowchart/diagram. *Note: Data were collected at the baseline, 3-month for all the allocated time periods, irrespective of whether the intervention had been initiated. 8month follow-up data was collected only till period 4. All 56 clusters were included for analyses.

**Fig 2:**
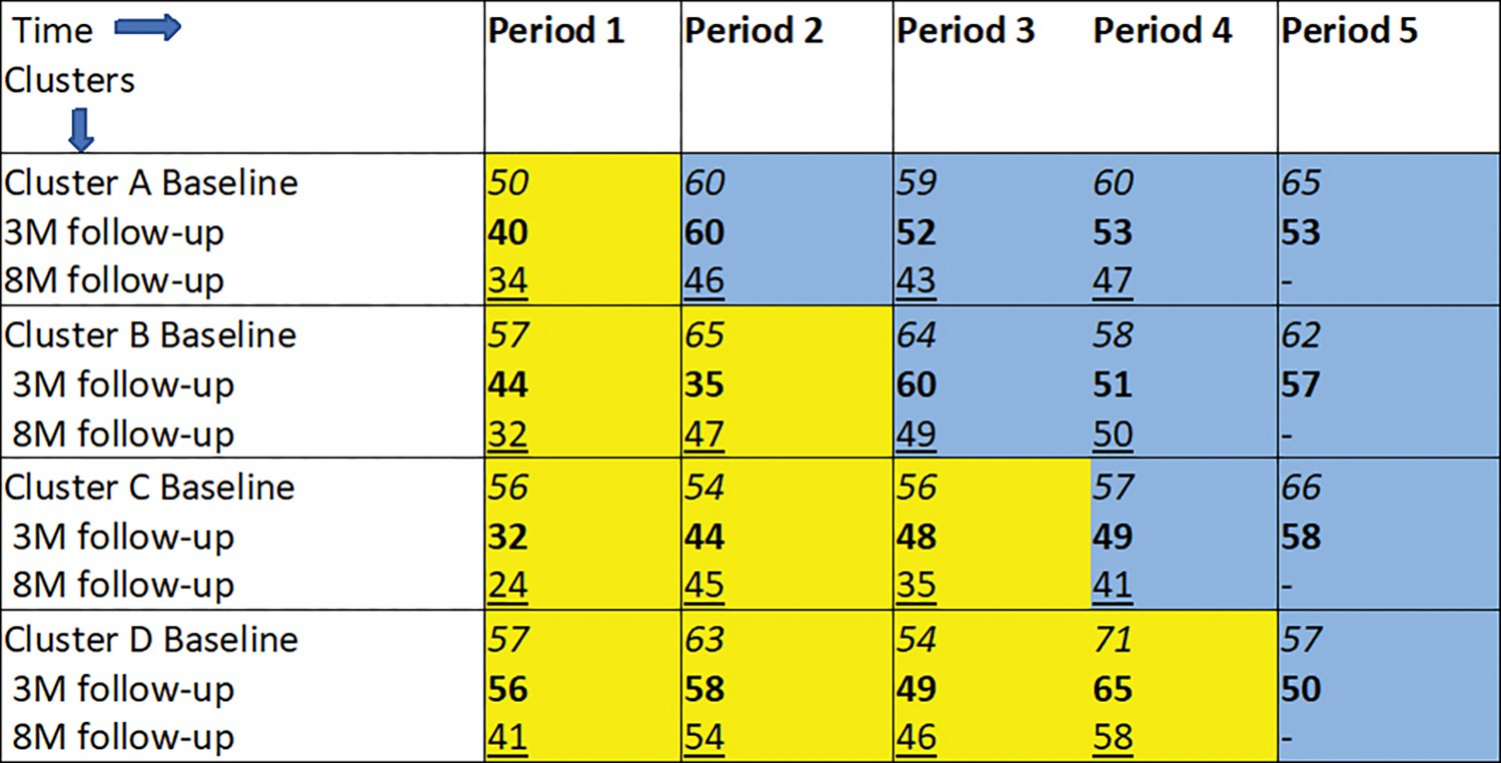
Stepped wedge design: Participants in each group of clusters and periods. *3M: 3-month follow-up; 8M: 8-month follow-up; 8M data was collected only till period 4. Different participants were recruited in each of the groups of clusters for each period; 3M and 8M follow-up measurements were completed on the same recruited participants. Control condition: yellow square. Intervention condition: blue square.

The stepped wedge trial followed a cross-sectional design [10, 11], with each 5-month period capturing different patients initiated in counseling and supports delivered by the Atmiyata champion (Fig 2). A detailed study protocol is described elsewhere [12]. The trial was conducted between April 2017 and August 2019, where villages (clusters) were allocated to four parallel groups based on geographical location. All four groups started at baseline in the control condition and the intervention was rolled out sequentially to each of the 4 groups in 5-month intervals (e.g., group B clusters transitioned to the intervention condition 5 months after group A). The trial duration was 25 months, including 5 months of baseline data collection in period one (Fig 1). All clusters received the intervention before the trial ended. The intervention roll-out was done in sequential phases. This was done to pace logistical processes and resources (e.g. research capacity, support from the local village leadership) needed for implementing the intervention in multiple villages, as the intervention could not be delivered in all clusters simultaneously.

### 2.2 Participants

Participants were adult community members residing in the Mehsana district with depression and anxiety symptoms. Inclusion criteria were persons aged 18–65 years with a score of ≥3 on the General Health Questionnaire (GHQ-12), indicating a ‘case’ of possible CMD. Exclusion criteria included people unable to provide informed consent, people with terminal medical conditions, and people presenting with suicidal ideation or suicidal behavior at the intake interview.

### 2.3 Randomization and treatment allocation

As primary health centers (PHC) in India serve distinct villages, we used PHCs to identify discrete geographical areas, consisting of all villages under a PHC, which was used as a unit of clustering for the trial. In Gujarat, how PHCs are distributed geographically limits the movement of people between PHCs, which helps to minimize contamination of the intervention A PHC in Gujarat covers, on average, a catchment area of 25000–30000 people which spans across 12–13 villages. At the time of the study, there were 56 PHCs in the Mehsana district, and we created 4 equal groups of 14 PHCs (A, B, C, D). These groups of clusters (A, B, C, D) were allocated to sequential steps in 5-month intervals. This study used a cross-sectional design, where outcome data is derived from different participants at each period. A random sample of 56 participants was selected from each cluster for each period. Details of blinding and contamination are mentioned elsewhere [12].

Champions were trained to identify people with CMD based on observed symptoms described by a participant during an unstructured interview. When a Champion identified a person, they thought had a CMD and intended to provide counseling sessions to the person, then they informed their supervisor and provided details of that person. The supervisor informed these details to the project manager, who created a screening list. Screening lists of each Champion were merged to create a master screening list, which formed the sampling frame for the intervention periods. Participants were randomly selected from the master screening list using a computer-generated random method. The trained data collection staff screened the participants using the GHQ-12. The participants who scored ≥3 were recruited for the trial until the target sample of 56 participants was reached for each group of clusters and each period.

Participants in the control condition were selected from electoral rolls using a systematic random sampling method with a pre-decided random start and random interval, with every *n*th number being selected by the statistician. Since the prevalence of CMD is 4–8% [1] a screening list of 1800 participants was prepared. For each group of clusters (A, B, C, and D) in each period, individuals in the screening list were screened to determine whether they constituted a ‘case’ of depression or anxiety, indicated by a GHQ-12 score of ≥ 3 until the target sample of 56 participants was reached for each group of clusters and each period [12].

### 2.4 Enhanced usual care (control period)

Enhanced Usual Care (EUC) was offered to all participants in the control condition who scored ≥3 on GHQ-12. EUC provided information on the impact of distress on physical and mental health and relevant information on available public mental health care services, including services by the District Mental Health Program (DMHP), and helplines for mental health support in and around the Mehsana district. The EUC also provided active support to participants in crisis. A crisis was defined as the participant revealing a recent self-harm attempt or expressing thoughts of self-harm during data collection. Such participants were encouraged to seek help immediately and data collectors sought participants’ consent to inform their family member or a friend about the crisis and mobilize social support to manage the crisis. This condition was considered as enhanced, as usual care provided in the community in these districts does not routinely provide information on mental health and distress or active support during a mental health crisis.

### 2.5 Intervention

The *Atmiyata* intervention consists of community volunteers involved in the identification and delivery of mental health and social care support to people with CMDs. The intervention has been described extensively elsewhere [12].

*Atmiyata* community volunteers (Champions) are trained to (i) identify persons with CMD and provide 4–6 counselling sessions; (ii) raise community awareness on social issues impacting mental health, by ‘narrow-casting’ four 10-minute films dubbed in *Gujarati on* commonly experienced social issues in the community (unemployment, family conflict, domestic violence, and alcoholism); (iii) refer cases of severe mental illness (SMD) to mental health services in the public health system and (iv) facilitate access to social benefits.

Champions receive 40 hours of training over 3 weeks, including practice-based counseling sessions. Champions are mentored and supervised by trained mental health professionals (called Community Facilitators) every fortnight. Each Community Facilitator is responsible for 40–50 Champions (S1 Fig).

Champions deliver counseling sessions over 6 to 12 weeks, with no set time between sessions. Sessions lasted 20–40 minutes, based on three evidence-based techniques used in other similar programs [3, 4]: active listening, behavior activation (activity scheduling), and problem-solving techniques.

The number of sessions delivered is left to the champion’s discretion and participant consent. All sessions were in the local (Gujarati) language and Champions were expected to deliver the sessions as per protocol. Champions delivered the intervention at the participant’s home or a location preferred by the participant (e.g. at the Champion’s home, a community place such as a village hall, temple, etc.). As this was an implementation research study, we wanted to assess the intervention as implemented in ‘real-life’ settings where the number, duration, and frequency of sessions will vary between the different provider (Champion) and participant dyad. Broad guidelines and recommendations were therefore provided on the number, duration, and frequency of sessions to Champions (e.g. a range of 4–6 sessions, duration from 20–40 min, and period of 6–12 weeks) which both mimics what is likely to happen in real-life clinical settings and allows exploration of dose-response effects.

### 2.6 Data collection timeline

Baseline data collection started in all groups of clusters in April 2017. The first cluster group (Group A) entered the intervention in September 2017 and the last (Group D) in December 2018.

### 2.7 Outcomes

The primary outcome was the proportion of participants with symptomatic improvement in CMD in the intervention condition compared to the control condition, measured by the validated Gujarati version of GHQ-12 at a 3-month follow-up. GHQ-12 scores were expressed as both a continuous outcome (with scores ranging from 0 to 12, higher scores indicating greater severity of symptoms) [13] and as a categorical outcome (case defined as ≥3 scores on GHQ -12; non-case as a score of less than 3) and an 8-month follow-up to evaluate sustained effects of the intervention.

Secondary outcomes include the accuracy of identification of CMD cases by champions and improvement in health-related quality of life using the EQ-5D [14]. Euro quality of life 5D (EQ-5D) is a standardized instrument that comprises five dimensions: mobility, self-care, usual activities, pain/discomfort, and anxiety/depression. Each of the 5 dimensions comprising the EQ-5D descriptive system is divided into 5 levels of perceived problems. Levels 1 to 5 indicate no problem, slight problem, moderate problem, severe problem, and extreme problem. A unique health state is defined by combining 1 level from each of the 5 dimensions. Each state is referred to in terms of a 5-digit code. These EQ-5D-5L health states, defined by the EQ-5D descriptive system were then converted into a single index value. Responses for all 5 dimensions were merged into a single index score [14]. Secondary outcomes also include improvement in psychiatric symptoms using the Self-report Questionnaire (SRQ) [15], improvement in depression and anxiety symptoms using the Patient Health Questionnaire (PHQ-9) [16], the Generalized Anxiety Disorder Scale (GAD-7) [17], functioning (WHO-DAS-12) [18] and improvement in social participation using the Social Participation Scale (SPS) [19]. User satisfaction with the intervention was assessed using the Client Satisfaction Questionnaire (CSQ) [20] only among intervention condition participants. All scales were translated to Gujarati and back-translated to English and validated before use. Details of all the tools are described in protocol paper [12].

Data on serious adverse events (defined as attempted suicide, self-harm, or death by suicide) was collected and reported to the ethical review committee in India (Indian Law Society Ethics Committee) and to the funding organization based on a protocol for reporting adverse events developed by the project team.

### 2.8 Statistical methods

#### 2.8.1 Sample size

The sample size was estimated using the stepped wedge function of STATA with the Hemming method [21]. The planned sample size of 1120 (56/clusters group/per period) was adequate to detect a 13% difference (constant time independent intra-cluster correlation (ICC) = 0.1), number of steps = 4, number of clusters randomized in each step k = 14, average cluster size m = 4, 80% power, and α = 0.05) in CMD recovery at 3-months follow-up between participants in intervention and control conditions. ICC was assumed to be 0.1 based on our previous study [8] also to confirm that we would have adequate power under the plausible circumstances for a cross-sectional SWCRCT likely to be more efficient [22].

#### 2.8.2 Statistical analyses

Intervention effects were estimated from the model using methods described by Hussey and Hughes [10]. Data were analyzed on an intention-to-treat basis, where participants were analyzed as they were allocated to the intervention. Baseline characteristics were summarized using counts (percentages) for categorical variables and means (SD) or median interquartile range (IQR) for continuous variables. All statistical analyses were carried out by means of multilevel mixed models containing a random intercept for cluster alongside the fixed effect of interest (intervention) and applicable covariates (period, age, gender, and baseline score of the respective outcome). Continuous outcomes were analysed by canonical linear mixed regression models (reported as adjusted mean differences, Cohen’s *d* and their respective 95% confidence intervals), while binary outcomes were analysed using logistic mixed regression models (reported as Odds Ratio’s and respective 95% confidence intervals). Missing data was assumed to be missing at random and hence no imputation was performed, since multilevel mixed models inherently handle such missing data appropriately for statistical inference.

Demographic variables were assessed cross-sectionally between arms only at baseline, while primary and secondary outcomes were assessed between arms cross-sectionally at baseline, at the 3-month follow-up and at the 8 month follow-up separately (i.e. no longitudinal analysis within clusters was performed), as described by Hemming *et al*. [22].

As pre-specified in the protocol, we also analyzed model extensions: 1) random cluster by period effect, 2) random cluster by treatment effect, and 3) Interaction between intervention and time period. Details are described in the supplementary material. The model extensions were used for secondary analyses purpose. Each model extension makes implicit modifications to the assumptions about the correlation structure.

STATA version 14 was used for all statistical analyses (StataCorp; College Station, TX). All results are reported as per the CONSORT extension for stepped wedge cluster randomized trial guidelines [23].

## 3. Results

A total of 1191 participants were recruited (583 started in the control condition and 608 started in the intervention condition) (Fig 1) into the trial. All participants allocated to the intervention condition received the counseling intervention. Outcome data were available for 471 control participants (19% lost to follow-up) and 543 intervention participants (11% lost to follow-up) at 3-month follow-up. Reasons for lost to follow-up (at 3-month) included the participant moving outside the trial district (n = 25,14.0%), long-term stay out of district for work or other reasons (n = 90, 50.80%), major illness (n = 7, 4.0%), refusal to participate in the follow-up assessment (n = 47, 26.6%) and other reasons (n = 8, 4.5%). For the intervention condition, 8 months of follow-up data for period 5 was not collected due to logistical constraints (Fig 2). At the 8-month follow-up, 416 participants started in the control condition and 276 participants started in the intervention condition. At follow-up periods, no participants displayed suicidal ideation, suicidal attempts, or self-harm.

Participant demographic characteristics at baseline are shown in Table 1. Compared to intervention participants, participants in the control condition were older, less educated, and more likely to be employed (Table 1).

**Table 1:** Baseline demographic characteristics of the participants by condition.

Demographic variables	Control condition (n = 583)	Intervention condition (n = 608)
Age (years), Median (IQR)	45 (18)	40 (18)
Sex, N (%)		
Male	249 (42 ∙8)	241 (39 ∙6)
Education (years of schooling)		
Median (IQR)	3 (8)	5 (9)
Marital Status, N (%)		
Currently married and living together	451 (77 ∙3)	407 (66 ∙8)
Married but separated	10 (1 ∙7)	31 (5 ∙1)
Divorced	8 (1 ∙4)	8 (1 ∙3)
Widowed	83 (14 ∙1)	112 (18 ∙4)
Never married	31 (5 ∙5)	51 (8 ∙4)
Employment status, N (%)		
Unemployed	146 (25)	206 (33 ∙8)
Religion, N (%)		
Hindu	551(94 ∙5)	595 (98)
Muslim	24 (4 ∙1)	11 (1 ∙9)
Caste, N (%)		
Upper Caste	135 (23 ∙1)	168 (27∙7)
Other Backward Caste	239 (40 ∙9)	314 (51∙8)
Scheduled Tribes	8 (1 ∙4)	6 (1)
Scheduled Caste	144 (24 ∙7)	111 (18∙3)
Don’t know	50 (8 ∙6)	6 (1)

Data are presented as total number (%); IQR Interquartile range

Baseline covariates for GHQ were adjusted in the final regression analysis as the mean scores for GHQ were significantly higher in the intervention condition compared to control (Table 2).

**Table 2:** Baseline differences between control and intervention condition participants using mixed models (adjusted for cluster and period).

Scales		Control condition (n = 583)	Intervention condition (n = 608)	Adjusted Mean difference (95% CL)	P value
General health questionnaire (GHQ-12)	Mean (SD)	6 ∙0 ± 2 ∙6	7 ∙7 ± 2 ∙7	1.14 (0.67 to 1.61)	0 ∙000*
	Median (Q1, Q3)	6 (4, 8)	8 (5, 10)		
Patient Health Questionnaire (PHQ-9)	Mean (SD)	12 ∙0 ±5 ∙6	14 ∙2 ± 5 ∙2	0.59 (-0.38 to 1.57)	0 ∙23 NS
	Median (Q1, Q3)	12 (8, 16)	14 (10, 18)		
Self-reporting questionnaire (SRQ-20)	Mean (SD)	14 ∙0 ± 4 ∙5	15 ∙0 ± 4 ∙0	-0.07(-0.84 to 0.70)	0 ∙85 NS
	Median (Q1, Q3)	15 (11, 18)	16 (12, 18)		
Generalized anxiety disorder (GAD-7)	Mean (SD)	10 ∙0 ±5 ∙0	11 ∙6 ± 4 ∙3	0.85 (-.04 to 1.75)	0 ∙06 NS
	Median (Q1, Q3)	10 (7, 13)	12 (8, 15)		
Social Participation scale (SPS)	Mean (SD)	20 ∙1 ± 18 ∙8	21 ∙5 ± 17 ∙8	-2.37 (-5.6 to 0.91)	0 ∙15 NS
	Median (Q1, Q3)	15 (6, 29)	18 (7, 31)		
Quality of life (EQ-5D)	Mean (SD)	0 ∙64 ± 0 ∙15	0 ∙58 ± 0 ∙16	-0.02 (-0.05 to 0.01)	0 ∙23 NS
	Median (Q1, Q3)	0 ∙66 (0 ∙54, 0 ∙75)	0 ∙62 (0 ∙50, 0 ∙69)		
WHO-Disability questionnaire (WHO-DAS)	Mean (SD)	24 ∙2 ± 8 ∙2	25 ∙7 ± 9 ∙04		0 ∙40 NS
	Median (Q1, Q3)	24 (18, 30)	25 (19, 31)	-0.64 (-2.1 to 0.88)	
W1 (no ∙ of days)	Mean (SD)	18 ∙2 ± 10 ∙5	22 ∙3 ± 8 ∙9	1.04 (-0.7 to 2.8)	0 ∙26 NS
	Median (Q1, Q3)	20 (10, 30)	30 (15, 30)		
W2 (no ∙ of days)	Mean (SD)	4 ∙8 ± 7 ∙0	6 ∙5 ± 9 ∙0	1.62 (0.28 to 2.9)	0 ∙01
	Median (Q1, Q3)	2 (0, 7)	3 (0, 10)		
W3 (no ∙ of days)	Mean (SD)	5 ∙7 ± 6 ∙9	8 ∙4 ± 9 ∙2	1.01 (-0.41 to 2.4)	0 ∙16 NS
	Median (Q1, Q3)	4 (0, 9)	5 (0, 12)		

Values are presented as mean ±SD; median (Q1, Q3); non-parametric Mann-Whitney Test was used to test significance ∙ NS: Not significant

**W1:** Overall, in the past 30 days, how many days were these difficulties (WHODAS scale) present? **W2:** In the past 30 days, for many days were you totally unable to carry out your usual activities or work because of any health conditions? **W3:** In the past 30 days, not counting the days that you were totally unable, for how many days did you cut back or reduce your usual activities or work because of any health condition?

Average GHQ scores were on par or above the recommended cut-off of 6 for primary-care attendees in India [24], and these community participants were not seeking any treatment in primary care. The mean score on the PHQ was 12.0 (SD = 5.6) and 14.2 (SD = 5.2) for control and intervention conditions respectively, indicating moderate depression. Mean scores on the GAD were 10.0 (SD = 5.0) and 11.6 (SD = 4.3) for the control and intervention conditions respectively, indicating moderate levels of anxiety. The average SRQ scores were high among both the intervention (mean = 14.0, SD = 4.5) and control condition participants (mean = 15.0, SD = 4.0) indicating a possible clinical diagnosis of a mental disorder.

Table 3 provides regression estimates for both primary and secondary outcomes using Generalized Linear Mixed Models. After adjusting for baseline covariates (GHQ-12), time points (periods), and cluster effects, categorical GHQ-12 recovery (a score of less than 3 at follow-up) was significantly higher among participants in the intervention condition at the end of 3 months (adjusted odds ratio OR 2.2; 95% CI 1.2 to 4.6). Goodness of fit for the logit mixed model has been reported as Akaike’s information criterion and Bayesian information criterion which are 833.47 and 872.79 respectively.

**Table 3:** Effect of intervention for primary and secondary outcomes using generalized linear mixed effect models.

	Control condition (n = 583)	Intervention condition (n = 608)	Regression estimates (95% CI)	P-value	ICC (95%CL)
**Primary outcome**	**Number**	**Number**	**Adjusted odds ratio**		
CMD participants improved at 3 months	76	102	2∙2 (1∙2 to 4∙6)	0∙01	0∙1 (0∙06 to 0∙3)
CMD participants improved at 8 months	77	125	3∙0 (1∙6 to 5∙9)	0∙001	0.2 (0.07 to 0.3)
**GHQ-12 on continuous scale**	**Median (Q1, Q3)**	**Median (Q1, Q3)**	**Adjusted mean difference (95% CI)**	**P value**	**Effect size**
GHQ-12 at 3 months	6 (3, 8)	5 (3, 8)	-0 ∙1 (-1 ∙6 to -0 ∙3)	0 ∙004	0 ∙2
GHQ-12 at 8 months	6 (3, 9)	3 (0, 6)	-2 ∙3 (-3 ∙1 to -1 ∙5)	0 ∙000	0 ∙5
**Secondary outcome at 3 months**	**Median (Q1, Q3)**	**Median (Q1, Q3)**	**Adjusted mean difference (95% CI)**	**Effect size**	
Patient Health questionnaire (PHQ-9)	11 (7, 15)	11 (6, 15)	-1.8 (-3.0 to -0.6)	0 ∙3	
Self-reporting questionnaire (SRQ-20)	14 (10, 18)	14 (8, 17)	-1.7 (-2.7 to -0.6)	0 ∙2	
Generalized anxiety disorder (GAD-7)	9 (6, 13)	8 (5, 12)	-0.8 (-1.9 to 0.3)	0 ∙2	
Social Participation scale (SPS)	14 (5, 26)	13 (3, 27)	-2.4 (-5.6 to 0.8)	0.04	
Quality of life (EQ-5D)	0 ∙64(0 ∙50, 0 ∙74)	0 ∙68 (0 ∙57, 0 ∙76)	0.03 (-0.01 to 0.7)	0 ∙2	
WHO-Disability questionnaire (WHO-DAS)	23 (18, 29)	22 (16, 28)	-1.4 (-3.0 to 0.09)	0.14	
W1 (no ∙ of days)	15 (10, 30)	15 (10, 30)	-0.3 (-2.3 to 1.7)	0.06	
W2 (no ∙ of days)	2 (0, 6)	1 (0, 7)	0 ∙07 (-0 ∙9 to 1 ∙1)	0.05	
W3 (no ∙ of days)	4 (1, 10)	5 (0, 10)	-0.3 (-1.6 to 1.1)	0.05	
**Secondary outcome at 8 months**	**Median (Q1, Q3)**	**Median (Q1, Q3)**	**Adjusted mean difference (95% CI)**	**Effect size**	
Patient Health questionnaire (PHQ-9)	12 (7 ∙7, 16)	6 (2, 11)	-4.4 (-5.7 to -3.0)	0 ∙6	
Self-reporting questionnaire (SRQ-20)	14 (10, 18)	0 (0, 10)	-3.6 (-4.9 to -2.3)	0 ∙4	
Generalized anxiety disorder (GAD-7)	10 (5, 14)	0 (0, 5)	-3 ∙6 (-4 ∙7 to -2 ∙4) -3.5 (-4.6 to -2.2)	0 ∙5	
Social Participation scale (SPS)	14 (3, 31)	7 (0, 22)	-6.4 (-9.8 to -3.0)	0 ∙3	
Quality of life (EQ-5D)	0 ∙67 (0 ∙56, 0 ∙75)	0 ∙73 (0 ∙59, 0 ∙84)	0 ∙06 (0 ∙03 to 0 ∙1)	0 ∙3	
WHO-Disability questionnaire (WHO-DAS)	23 (18, 29)	17 (13, 26)	-3.6 (-5.3 to -2.0)	0 ∙4	
W1 (no ∙ of days)	15 (10 ∙ 30)	10 (3, 30)	-3 ∙2 (-5 ∙9 to -0 ∙4)	0 ∙2	
W2 (no ∙ of days)	3 (0, 10)	0 (0, 5)	-3 ∙0 (-4 ∙5 to -1 ∙4)	0 ∙3	
W3 (no ∙ of days)	5 (0, 10)	4 (0, 10)	-2.8 (-4.7 to -0.9)	0 ∙3	

Adjusted estimates are presented as Odds ratio for primary outcome on binary scale and adjusted mean difference for secondary outcomes on continuous scales using a generalised linear mixed model and are adjusted for, random effect of clustering and fixed effect of underlying temporal trends. NS: Not significant

**W1:** Overall, in the past 30 days, how many days were these difficulties (WHODAS scale) present? **W2:** In the past 30 days, for many days were you totally unable to carry out your usual activities or work because of any health conditions? **W3:** In the past 30 days, not counting the days that you were totally unable, for how many days did you cut back or reduce your usual activities or work because of any health condition?

These effects were sustained at 8 months (adjusted OR 3.0; 95% CI 1.6 to 5.9). Similarly, continuous GHQ-12 scores were significantly lower among participants in the intervention condition at the end of 3 months (adjusted mean difference (AMD): -0.1; 95% CI: -1.6 to -0.3; effect size 0.2) and 8 months (AMD: -2.3; 95% CI: -3.1 to -1.5; effect size 0.5) (Table 3) ICC for the generalized linear mixed model on GHQ-12 continuous scale was 0.02 (0.003–0.10). Goodness of fit for the linear mixed model has been reported as Akaike’s information criterion and Bayesian information criterion which are 5059.46 and 5103.71 respectively.

We performed several additional model extensions. First, when allowing for different time points across clusters, the estimated treatment effect increased (S1 Table) and the correlation between observations in different periods was slightly lower than between observations within the same time points (period), indicating a slight decay of the correlation structure. Second, when testing for treatment effect heterogeneity, the intervention estimate increased compared to the base model, with similar correlations between observations in the intervention condition and control condition. (S1 Table). There were no significant differences (p<0.05) when allowing for interaction between intervention and time period (S2 Table).

### 3.1 Secondary outcomes

#### Identification and treatment of depression and anxiety symptoms

The champions’ case detection rate (accurate identification of common mental disorders) as compared to actual GHQ-12 scores (cut-off score of 3 or more) was 58% for all the clusters and periods in the intervention condition.

Data on counseling sessions attended was available for 89% (N = 543) of the intervention sample. Participants attended a mean of 5.2 (SD = 1.2) and a median of 6 (Q1, Q3: 5, 6) sessions. At the 3-month follow-up, 2% (n = 11) of participants were referred to public health services (either the PHC or the DMHP) by the champions. In the control group, 52% (n = 244) were similarly referred to public health services.

PHQ-9 depression scores were significantly lower among intervention condition participants compared to those in the control condition, with an adjusted mean difference (AMD) of -1.8 (95%CI: -3.0 to -0.6; effect size 0.3) at 3 months and AMD of -4.4 (95% CI: -5.7 to -3.0; effect size 0.6) at 8 months (p<0.05). PHQ-9 scores among intervention condition participants changed from moderate depression scores of 10–12 at baseline to mild depression scores of 5–9 (range of adjusted means) at 8-month follow-up (p<0.05). On anxiety symptoms as measured by the GAD-7, the intervention group did not show improvement (AMD: -0.8 (-1.9 to 0.3; effect size 0.1) at 3 months whereas showed significant improvement -3.5 (-4.6 to -2.2; effect size 0.5) at 8 months follow-up (p<0.05). Among participants in the intervention condition, GAD scores changed from moderate anxiety symptoms at baseline to mild to none at 8-month follow-up. There was no significant improvement at the 3-month follow-up on functioning scores, as assessed by the WHO-DAS whereas showed improvement at 8 month (AMD of -3.6 (95%CI: -5.3 to -2.0; effect size 0.4). WHO-DAS scores increased significantly at 8 months follow-up in intervention participants with AMD of -3.2 (-5.9 to -0.4 effect size 0.2), -3.0 (-4.5 to -1.4; effect size 0.3) and -2.8 (-4.7 to -0.9; effect size 0.3) for W1, W2, W3 respectively. Compared to baseline, SRQ scores improved at 3 month (AMD -1.7 (95%CI: -2.7 to -0.6; effect size 0.2) and at 8-month follow-up (AMD: -3.6 (95%CI: -4.9 to -2.3; effect size 0.4). Health-related quality of life assessed by EQ-5D, showed significant improvement 0.06 (95%CI: 0.03 to 0.1; effect size 0.3) at 8 month follow-up (Table 3).

On the Client Satisfaction Questionnaire, the average score was 24.0 ±5.2 demonstrating a high satisfaction level with *Atmiyata* intervention at the end of 8 months.

Considering the loss to follow-up rate was greater than 10% at the end of 3 months follow-up and 8 months follow-up, baseline measures between the participants who continued the intervention and those lost to follow-up in each condition were analyzed separately. For data at 3 months follow-up in the intervention condition, no significant differences were observed between the baseline values on the GHQ-12, PHQ-9, GAD-7, and SRQ-20) among participants who remained in the intervention compared to those lost to follow-up. In the control condition, the baseline values were significantly higher among participants who remained in the intervention (p<0.05).

At 8-month follow-up, mean baseline values of measures were similar (p>0.05) in those who remained in the trial versus those lost to follow-up in both control and intervention conditions.

Moreover, the power to detect observed differences between the intervention and control condition remained at 80% at the end of 3 months. For an 8-month follow-up, data were considered until period 4 so based on the observed difference with the sample size of 276 in intervention and 217 in control with 3 steps and 48 clusters the power remained 80%.

The overall results across primary and secondary outcomes demonstrate that the intervention was effective, with effects sustained and improving over time, including at 8 months follow-up after counseling sessions ended.

## 4. Discussion

To our knowledge, this is the first study to systematically evaluate the large-scale implementation of a trained community volunteer-led intervention for common mental disorders using a pragmatic implementation research design in a low and middle-income country. Our findings show that the *Atmiyata* intervention is effective in reducing symptoms of common mental disorders and effects are sustained over time. Intervention participants with a common mental disorder were twice as likely to improve at a 3-month follow-up and three times more likely to improve at an 8-month follow-up compared to individuals receiving enhanced usual care. The intervention was also effective in reducing symptoms of depression and anxiety and improving quality of life at 3 months, and improving social participation and improving functioning at 8-month follow-up. In addition, *Atmiyata* Champions were reasonably skilled (58 percent specificity) at identifying persons with a common mental disorder compared to a structured measurement instrument (GHQ-12) administered by research staff.

Our data shows that improvements in the primary outcome are enhanced over time, as indicated by the odds of improvement of depression and anxiety symptoms at 8 months follow-up being higher than at 3-month follow-up. Counseling sessions were completed before the 3-month follow-up data collection and no additional sessions were delivered afterward. This may suggest the intervention’s benefits continue even after the counseling sessions end. As expected, improvement in social functioning and reduction in disability is seen at 8 months but absent at 3 months, suggesting a lag between symptomatic improvement and functional improvement.

The *Atmiyata* intervention used community volunteers as compared to many other studies or programs of NSHW interventions which use either paid lay counselors or public health community workers to deliver mental health interventions. From a health system perspective, the effectiveness of community volunteers in improving depression and anxiety symptoms, as shown in this trial, is important for several reasons. First, in most LMICs, primary healthcare staff are overburdened with multiple health tasks and may lack the capacity to take on additional mental health tasks. Our intervention taps into existing community social capital and increases the pool of people available to deliver mental health interventions. Second, using community volunteers and a social model of mental distress to explain symptoms improved the acceptability of the intervention and reduced the stigma associated with seeking help for mental health problems. Third, the Atmiyata intervention included participants who had symptoms of depression or anxiety and required support but were not seeking or receiving care at PHC facilities. Studies have also found that most people seeking help in PHC for physical health symptoms displayed somatic symptoms, had low awareness of depression and few had a previous diagnosis of depression and/or received psychological treatments or anti-depressants [25, 26]. The *Atmiyata* intervention locates distress in the context of difficulties in day-to-day living and social interactions rather than using a bio-medical health paradigm for understanding mental health distress. In our experience, we find this paradigm is closely aligned with the participants’ explanatory models and thus improves the acceptability and adherence to the intervention offered by *Atmiyata* Champions. Finally, using community volunteers reduces the intervention cost and thus, improves the sustainability of the intervention. Atmiyata is currently examining the feasibility and effectiveness of additional specific intervention components where there is significant community demand for support) and for specific sub-groups (e.g. women with post-partum depression). Additional funding has been secured to continue the *Atmiyata* program in the Mehsana district for three more years. We have also initiated a program of arranging local site visits for civil society organizations and policymakers across the country to interact with beneficiaries and gain first-hand experience of the effectiveness of the program. We plan to launch an online training module for policymakers to learn more about the *Atmiyata* program and encourage widespread adoption in diverse communities in India and other LMICs.

There are several challenges to implementing an intervention using community volunteers. First, any training or capacity building needs to emphasize the need to maintain confidentiality, especially when such volunteers are drawn from the same community where they are likely to have more than a passing acquaintance with those seeking help. Other challenges include identifying suitable volunteers and maintaining their motivation over time. The *Atmiyata* program developed and used manuals outlining criteria to assess the suitability of potential Champions when selecting volunteers. There is also significant emphasis on activities to retain the motivation of volunteers including weekly and monthly support meetings and public recognition of their contribution to society.

## 5. Conclusion

The *Atmiyata* intervention provides evidence of the effectiveness of a non-specialist delivered mental health intervention for improving symptoms related to common mental disorders in an LMIC setting. This study contributes to the scarce implementation research evidence base for NSHW interventions in LMICs. The findings and the process of implementation of this intervention may support policymakers in India and other countries in decision-making related to what type of interventions to employ for addressing depression and anxiety in their context.

## 6. Limitations

There are several limitations of this study. Not everyone improved; even at the end of 8 months, nearly 30% of those who received the intervention had significant symptoms of depression and anxiety. The *Atmiyata* intervention may therefore not be a suitable standalone intervention but better placed as a core component of informal community care, with close linkages to primary and secondary mental health care for addressing more specialized care needs. Although a stepped wedge design has advantages over a parallel cluster design specifically related to constraints related to resources and the challenge of getting a complex intervention ready for implementation, it is challenging to ensure all the clusters are ready to implement on schedule, complicated to analyze as it requires certain assumptions and is dependent to time points if outcomes are already improving. However, the large sample size and a large number of clusters and steps in this trial allowed us to estimate and adjust for time trends. Another challenge is maintaining treatment fidelity when using community volunteers. Further, participants in this trial are community members who are not necessarily seeking treatment from a health provider and thus may be different in some respects from those presenting for healthcare at primary care clinics. We did not conduct a diagnostic interview at baseline (the gold standard) but used a locally validated screening tool (GHQ-12) and locally validated cut-off measures to determine ‘caseness’, or the presence of a common mental disorder [12]. While Endsley et al. [27] recommended a cut-off score of 2 and above for community-based studies for the identification of common mental disorders, we took a conservative approach and used the Goldberg et al. [13] criteria (score of 3 and above) for identifying those with common mental disorders. Although we used the 3 cut-offs on GHQ-12 for case identification, the median scores on GHQ-12 were 6 and 8 respectively for participants in the control and intervention condition which are recommended cut-offs for identifying individuals with common mental disorders in primary-care attendees [24]. It is important to note that there was a significant difference in baseline scores on various scales GHQ-12, PHQ-9, and GAD-7 between participants in the intervention and control conditions. Participants in the intervention condition are randomly recruited from a list of people identified by *Atmiyata* Champions as having a common mental disorder as compared to control participants, who were identified using the GHQ-12 as a screening instrument with randomly selected subjects from an electoral register. We interpret these differences at baseline as evidence that Champions are accurately identifying persons with significant mental health problems (moderate depression and anxiety), which may provide indirect evidence of the efficacy of training received. These baseline differences were adjusted during analysis. Despite the limitations, the pragmatic nature of this trial and limited exclusion criteria (reflecting real-world conditions) enhances the generalizability of the findings.

## 7. Future directions

This study highlights several future research and practice directions. First, more evidence generated through pragmatic trial designs such as stepped wedge trials is needed in LMICs. Second, more evidence on the sustained implementation of community-volunteer-led models is needed, particularly evidence or models that consider aspects that impact sustainability such as incentives for community volunteers, waning motivation levels over time, and evolution of social dynamics in villages. Related to sustainability, Atmiyata is testing whether a social franchising model [28] might be an effective model for maintaining the intervention at scale. Third, mental health is shaped by many social, cultural, and economic factors, and it may be relevant for future research to examine whether specific intervention components (e.g. addressing interpersonal violence) may be beneficial to introduce to an intervention like Atmiyata.

## 8. Supplementary material (statistical details of model extensions)

Correlation structures in an SW-CRCT design are more complicated than a parallel CRT where measurements are taken at a single cross-section only. While Hussey and Hughes’s model is commonly used for the analysis of SW-CRCT trials, it makes several assumptions such as the same underlying time trend across all the clusters, exchangeable or uniform correlation structure (observations within any cluster share the same correlation structure irrespective of how far apart in time measurement are taken) and intra-cluster correlation (ICC) is independent of time period of measurement. However, these assumptions may be untenable, and correlations might decay with increasing separation in time between observations, which can be studied in the alternative discrete-time decay model by Hooper (model extension 1). Thus, to handle the complex correlation structure in our study we performed model extensions.

Our study may be regarded as a multi-centric study as there are many clusters and each cluster is exposed and unexposed to intervention therefore, treatment-effect heterogeneity can be investigated by including treatment by cluster interaction (model extension 2) This model allows the variability between clusters to differ between intervention and control periods. In addition to treatment heterogeneity across clusters, treatment effect might vary with time as SW-CRCT runs over multiple time periods and can be investigated using a treatment-by-time interaction (model extension 3). This model explores the interaction between intervention and time period, and modifies fixed effect components only with no modifications to the correlation structure. We present data on these model extensions exploring the sensitivities to deviations from the basic assumptions in Hughes and Hussey’s model around time points, treatment effect, and correlation structure.

## Supporting information

S1 Checklist CONSORT 2010 checklist of information to include when reporting a randomised trial*. (DOC)

S1 Fig *Atmiyata* implementation team structure. (TIF)

S1 File (XLSX)

S1 Table Model extensions for primary outcome. (DOCX)

S2 Table Model extension 3, for interaction between intervention and time period. (DOCX)
