# Supplementary figures and images for: Atmiyata, a community champion led psychosocial intervention for common mental disorders: A stepped wedge cluster randomized controlled trial in rural Gujarat, India
Source: PLoS One. 2023 Jun 8;18(6):e0285385. doi: 10.1371/journal.pone.0285385 (PMC10249851; doi:10.1371/journal.pone.0285385)

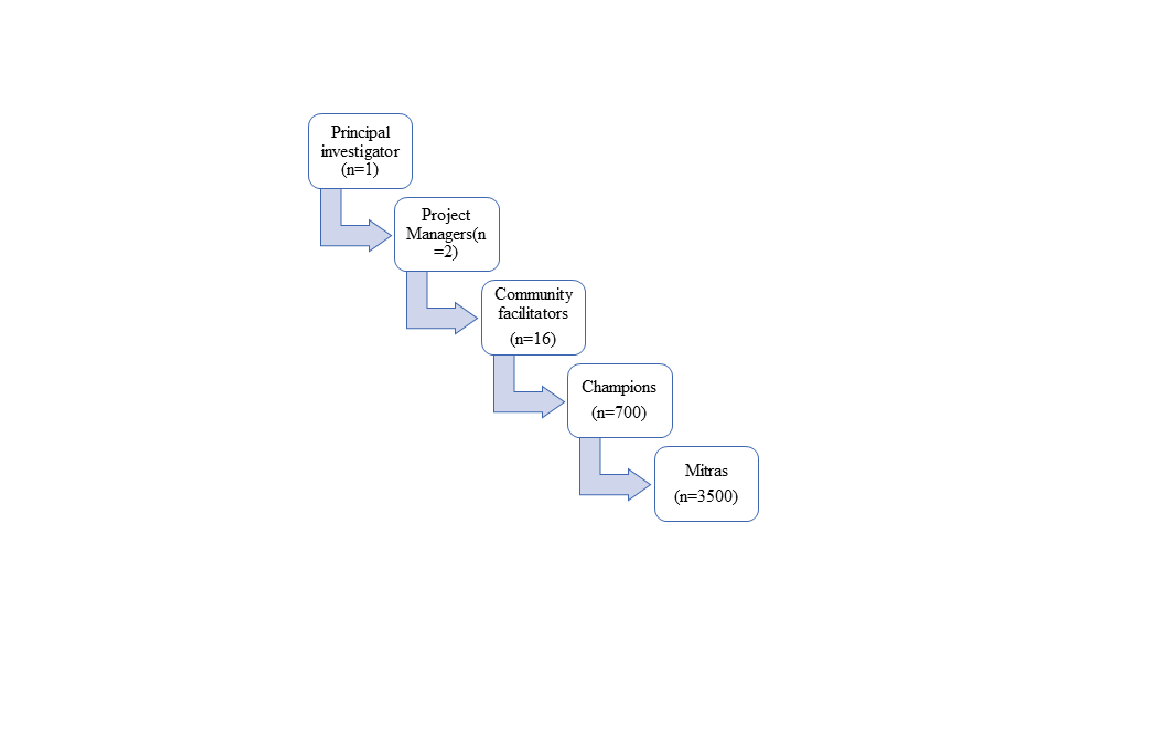

Supplement: S1 Fig — (TIF) [file pone.0285385.s002.tif]
