# Supplementary material for: Atmiyata, a community champion led psychosocial intervention for common mental disorders: A stepped wedge cluster randomized controlled trial in rural Gujarat, India
Source: PLoS One. 2023 Jun 8;18(6):e0285385. doi: 10.1371/journal.pone.0285385 (PMC10249851; doi:10.1371/journal.pone.0285385)
Supplement: S1 Table — (DOCX) [file pone.0285385.s004.docx]

**S1 Table: Model extensions for primary outcome**

| **Model** | **Intervention effect (ODDs ratio)** **(95% CI)** | **Within**  **period ICC** | **Between period ICC** | **Control ICC** | **Intervention**  **ICC** |
| --- | --- | --- | --- | --- | --- |
| Random cluster effect | 2∙2 (1∙2 to 4∙6) | 0∙1 (0∙06 to 0∙3) |  |  |  |
| + Random cluster by period effect (extension 1) | 3∙4 (1∙5 to 8∙1) | 0∙1 (0∙09 to 0∙3) | 0∙09 (0∙02 to 0∙3) |  |  |
| + + Random cluster by treatment effect (extension 2) | 3∙5 (1∙7 to 8∙8) |  |  | 0∙3 | 0∙3 |

*All models are adjusted for clustering*

*+ ICC presented is within same cluster same period and same cluster different period*

*++ ICC presented is within same cluster both treated; and same cluster both untreated (control)*

*All the ICC are presented on logistic scale*
