# Supplementary material for: Atmiyata, a community champion led psychosocial intervention for common mental disorders: A stepped wedge cluster randomized controlled trial in rural Gujarat, India
Source: PLoS One. 2023 Jun 8;18(6):e0285385. doi: 10.1371/journal.pone.0285385 (PMC10249851; doi:10.1371/journal.pone.0285385)
Supplement: S2 Table — (DOCX) [file pone.0285385.s005.docx]

**S2 Table: Model extension 3, for interaction between intervention and time period**

| **Overall time on treatment effect** | **Odds Ratio** | **95% CL** | **P-value** |
| --- | --- | --- | --- |
| 2 Period on intervention | 0∙96 | 0∙43-2∙1 | 0∙928 |
| 3 Period on intervention | 0∙58 | 0∙28-1∙21 | 0∙153 |
| 4 Period on intervention | 1∙05 | 0∙56-1∙95 | 0∙868 |
| 5 Period on intervention | 0∙84 | 0∙48-1∙4 | 0∙574 |

*Model adjusted for clustering. Estimates are presented as Odds ratio (95% CI)*
